# Supplementary material for: Nanopatterned Back-Reflector with Engineered Near-Field/Far-Field Light Scattering for Enhanced Light Trapping in Silicon-Based Multijunction Solar Cells
Source: ACS Photonics. 2023 Oct 26;10(11):4061–70. doi: 10.1021/acsphotonics.3c01124 (PMC10655497; doi:10.1021/acsphotonics.3c01124)
Supplement: Supplementary file 1 — ph3c01124_si_001.pdf [file ph3c01124_si_001.pdf]

Supplementary Materials: Nano-patterned back-reflector with engineered near-field/far-field light scattering for enhanced light trapping in silicon-based multi-junction solar cells

Andrea Cordaro[1,2,*], Ralph Müller[3], Stefan Tabernig[1,2], Nico Tucher[3], Patrick Schygulla[3], Oliver Höhn[3], Benedikt Bläsi[3], and Albert Polman[2]

[1]Van der Waals-Zeeman Institute, Institute of Physics, University of Amsterdam Science Park 904, 1098 XH Amsterdam, The Netherlands
[2]Center for Nanophotonics, AMOLF Science Park 104, 1098 XG Amsterdam, The Netherlands
[3]Fraunhofer ISE, Heidenhofstr. 2, 79110 Freiburg, Germany
[*]a.cordaro@amolf.nl

1 Theory and Design

The distribution of reflected power to the different diffraction channels (averaged over the simulation wavelength range and over both polarizations) for the optimal design is shown in Fig.1 of the main text. Most of the light reaching the back-reflector at the bottom of the Si cell is reflected equally to 6 diffraction channels beyond the critical angle for the Si/air interface with a $\simeq 15.4\%$ efficiency for each channel. It is interesting to point out that, while the FOM is polarization independent the coupling efficiencies to the channels at an angle are not (see Fig. S1a-b). Indeed, at normal incidence and for a normally reflected wave (e.g. the 0th diffraction order) the array is invariant under $\pi/3$ rotations. This implies that any in-plane polarization vector can be written as a linear combination of two basis vectors that span the entire plane and for which the response is identical by symmetry (e.g. the primitive translation vectors of the hexagonal lattice that form an angle of $2\pi/3$ and are of equal length). On the other hand, for light impinging from an angle, and hence by reciprocity reflecting at an angle, the lattice would look distorted and not highly symmetric, therefore the response is polarization dependent.

To understand why it is possible to achieve such a low FOM and to unravel the mechanism behind the back-reflector operation, it is useful to analyze the parameter space beyond the optimum values. Figure 2 a-b of the main text shows the reflectance to the 0th diffraction order (a) and parasitic absorption (b), averaged in the range 1000-1200 nm, as a function of nano–disk height and periodicity if the lattice fill factor is kept constant ($p/r = 3$) and close to that of the optimal grating. Next, taking cross-cuts of the data at different periodicities it is possible to analyze the reflectance to 0th–order and the parasitic absorption as a function of wavelength. It is worth highlighting a faint region around $p = 600$ nm where parasitic absorption is higher. To further explore the physical mechanism behind this slight increase, Fig. S2a-b displays the reflectance to 0th–order and the parasitic absorption as a function of wavelength for an array with $p = 600$ nm (and consequently $r = 200$ nm). The clearly visible sharp vertical feature in the reflectance spectra (panel a) corresponds to similarly sharp absorption peaks (panel b). This is due to the onset of higher diffraction modes that open up at grazing angles hence interacting more strongly with the nano-disks array and thereby inducing more losses. This phenomenon is known as Rayleigh anomaly (RA)[1,2].

To further test the applicability of Eq. 1 of the main text, Fig. S3a-b demonstrates how the 0th–order reflectance depends on the array periodicity and nano–disks radii and consequently on the fill factor F. Again, the general trend in reflectance is well captured by the simple model described in the main text with a small discrepancy due to resonances that cause a slight mismatch in the minima locations (green dashed line) in panels (a) and (b).

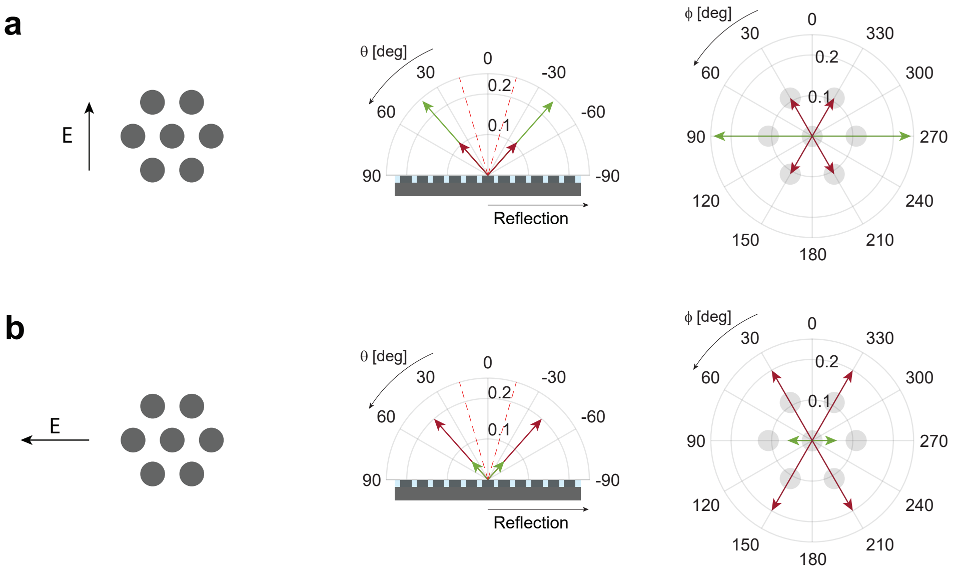

Figure S1. **a-b** Polar plots showing the fraction of incident power reflected (radial coordinate) to each diffraction channel (angular coordinates θ and ϕ) for for x- and y-polarized incident light averaged in the range 1000-1200 nm.

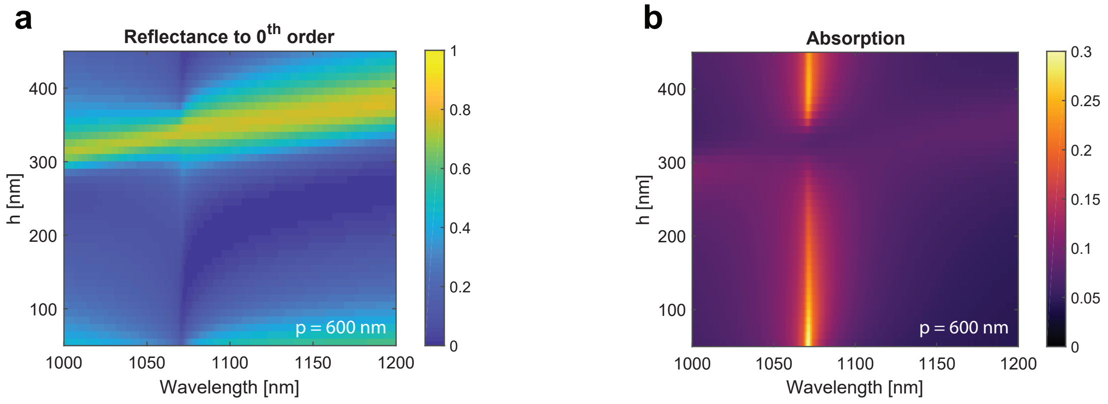

Figure S2. **a** Reflectance to the 0th diffraction order as a function of wavelength and nano–disk height for a fixed periodicity $p = 600$ nm. **b** Parasitic absorption for the same parameter space.

Understandably, there is an optimum fill factor to minimize reflection given the need of balancing the contributions from r_{Ag} and r_{FP}. It is worth pointing out that, different from other light redirection strategies employing gratings[3,4], in our case light is not steered at an angle by engineering the scattering of the single inclusion via resonances. On the contrary, sharp resonant modes should be avoided as they would result in severe parasitic loss. Our design redirects light at an angle by suppressing one of the available channels (0th–order) essentially by destructive interference. Therefore, parasitic absorption in the metal can still be mitigated.

2 Fabrication imperfections

As shown in Fig.4b of the main text, Ag sputtering on the pattern PMMA results in conically shaped air pockets included in the metal. To assess the impact of these fabrication imperfections on the optical performances of the grating we model these air inclusions as cones of air with a height of 500 nm (estimated from FIB cross-sections), angular aperture α, and distance to the Si substrate z. Figure S4 shows the simulated FOM $=$ Abs $+$ $R0^{th}$ (averaged

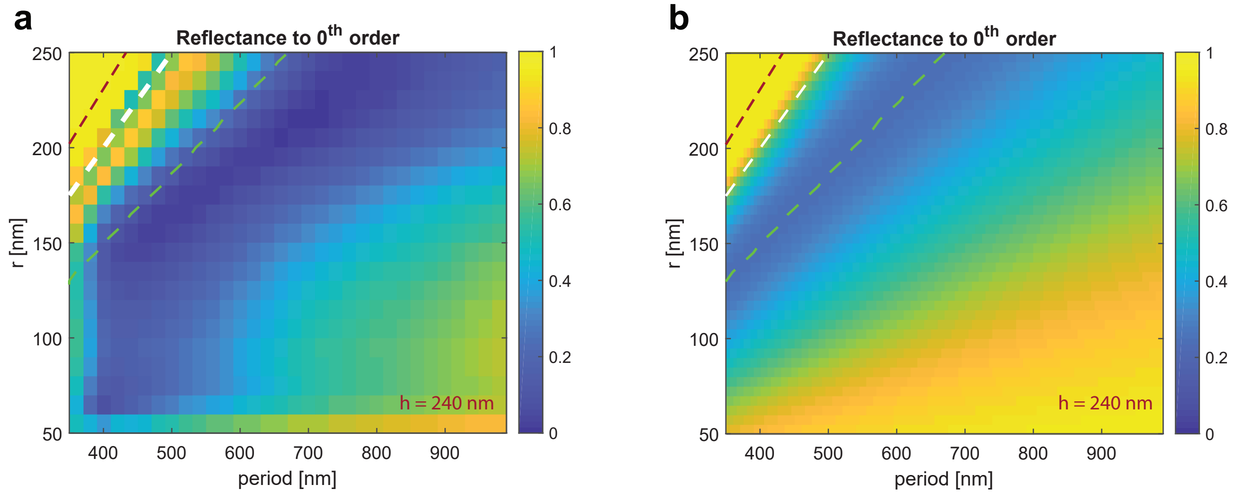

Figure S3. **a-b** Simulated (a) and modeled (b) reflectance to the 0th diffraction order, averaged in the range 1000–1200 nm, as a function of nano–disk radius and periodicity for a fixed nano-disk height $h = 240$ nm. The white and red dashed lines indicate the fill factors values at which nano–disks are touching each other and fully covering the substrate respectively. The green dashed line indicates the minima in panel b.

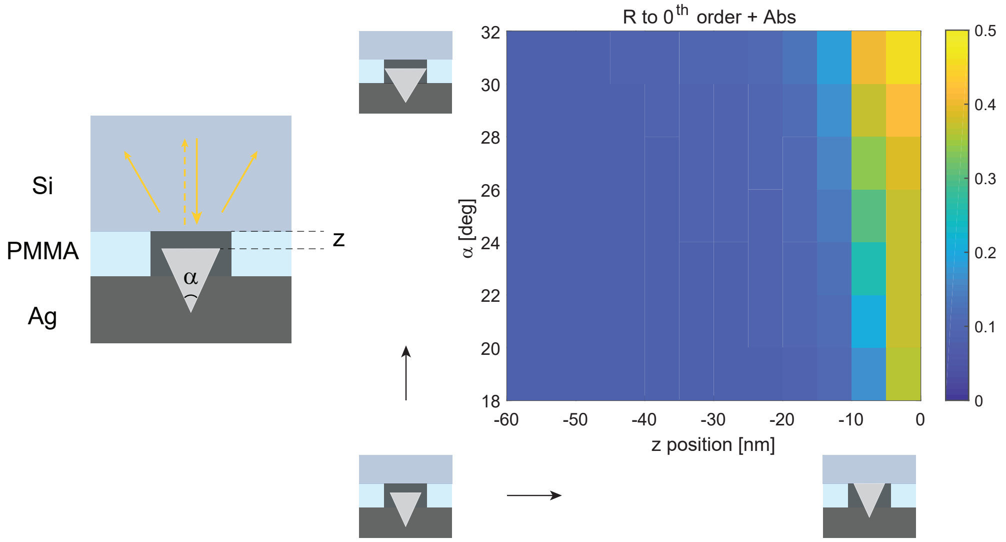

Figure S4. Simulated FOM $= \text{Abs} + \text{R0}^{th}$, averaged in the range 1000–1200 nm, as a function of the air inclusion angular aperture α and distance to the Si substrate z, as schematically depicted in the inset.

in the range 1000-1200 nm) as a function of α and z. The air inclusion position z has a big impact on the 0th–order reflectance and parasitic absorption for $z > -20$ nm, close to the skin depth of Ag in the considered wavelength range. On the other hand, α has much less influence on the FOM even in the extreme case of the cone base radius being equal to the nanodisk radius. From FIB cross-sections such that shown in Fig.4b of the main text, we estimated $\alpha \sim 28$ deg. and $z < -40$ nm. For such values the FOM is essentially unaltered.

3 Extended data

To demonstrate consistency and reproducibitly of the results, Figure S5 displays the optical and electronic characterization of a different cell from the same wafer (X702) as C1 of the main text and of a cell from a different wafer

(X629). Both wafers were processed in parallel and with the same fabrication procedure as described in the Methods section. Calibrated one-sun current-voltage measurements were performed also on these cells at ISE CalLab and the power conversion efficiency η, short circuit current density J_{SC}, open circuit voltage V_{OC}, and fill factor FF are summarized in the insets of Fig. S5b-d. Specifically, Cell C5 (wafer X702) shows the highest power conversion efficiency $\eta = 35.6\%$ while Cell C6 (wafer X629) shows the highest current gain in the Si sub-cell $+1.52$ $^{mA}/_{cm^2}$. This last result is compatible with that shown in Fig. 5a of the main text obtained for single-junction Si bottom cells.

In conclusion, it is important to compare the grating design presented here to the grating geometry[5,6] used in the current world record with a power conversion efficiency of η =35.9%. OPTOS calculations and experimental validation on Si bottom cells were performed for both grating designs (see Fig. S6). The outcome of the OPTOS calculation, shown in panels a-b, demonstrates a clear enhancement in the Si sub-cell absorption of both grating designs due to light trapping with a distinct advantage for the hexagonal design discussed here. Even though the total reflectance of the two designs is comparable, a lower parasitic absorption favors the AMOLF design. This comparison is validated experimentally by the measured EQE spectra of Si single-junction bottom cells. Also experimentally, the new design presented here shows superior performance compared to that used in the current world record. Corroborating the EQE spectra, the lower reflection achieved by the hexagonal grating can be ascribed to enhanced light trapping capabilities. The higher reflection and lower EQE compared to Fig. 5 of the main text are due to the absence of an anti-reflection coating at the top side of the cell.

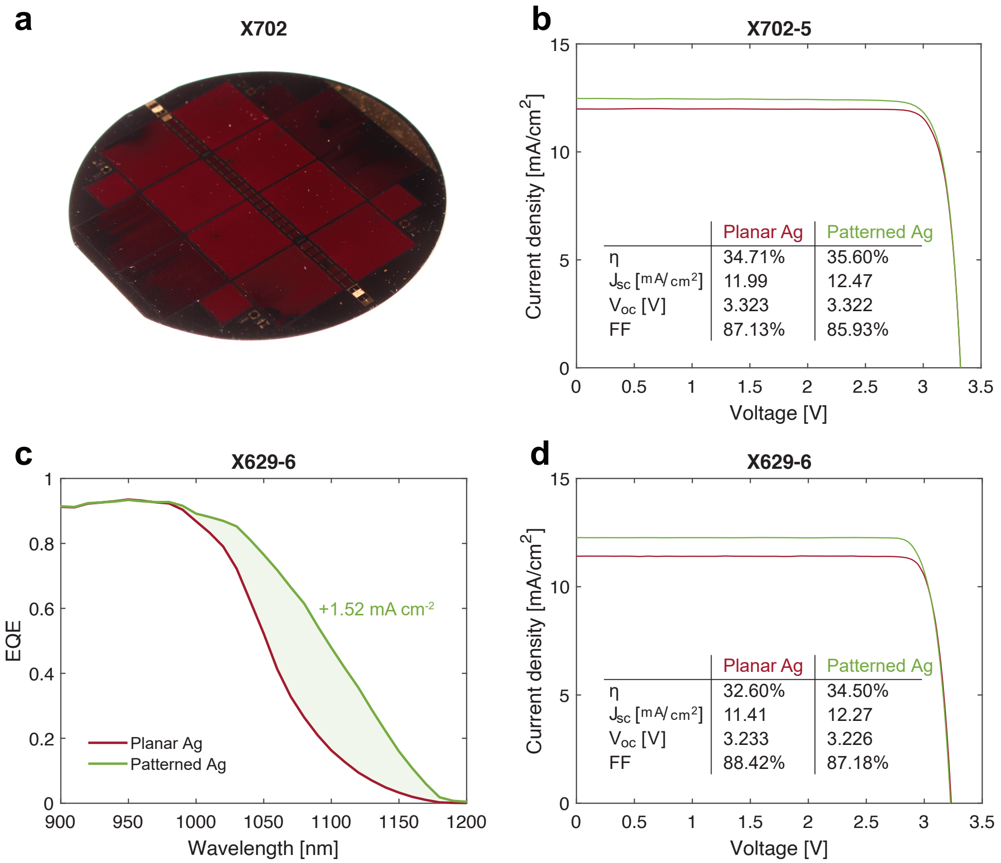

Figure S5. **a** Photograph of the front-side of the 4” wafer X702 containing the triple-junction Cell C1 of the main text and Cell C5 shown here. **b** Measured one-sun I-V characteristics comparing the Cell C5 (wafer X702) with the optimized hexagonal grating and its planar reference. **c** Experimental EQE spectra of the Si sub-cell (Cell C6 - wafer X629) for a patterned and planar back-reflector. **d** Measured one-sun I-V characteristics comparing the Cell C6 (wafer X629) with the optimized hexagonal grating and its planar reference.
Power conversion efficiency η, short circuit current density J_{SC}, open circuit voltage V_{OC}, and fill factor FF are summarized in the insets of panels b-d.

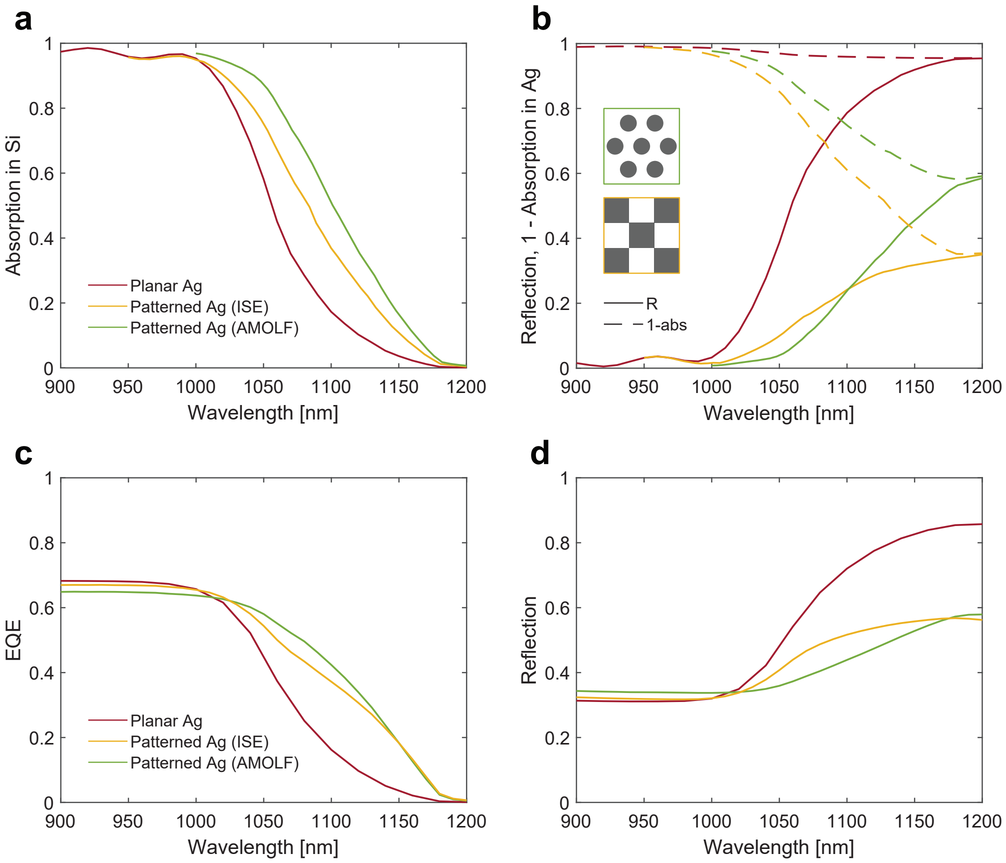

Figure S6. OPTOS calculated absorption in Si (**a**), reflectance, and parasitic absorption (**b**) spectra for the optimized design described above ("Patterned Ag (AMOLF)"), for the optimized design in Ref.[5] ("Patterned Ag (ISE)") and for a cell with a planar Ag back–reflector. The calculation assumes a double anti–reflection coating and a cell Si thickness of 280 μm. Inset: schematic of the two back-reflector geometries. Experimental EQE (**c**) and reflection (**d**) spectra for the AMOLF grating design, for the ISE design, and for planar Ag back–reflector.

References

1. Strutt, J. W. III. Note on the remarkable case of diffraction spectra described by Prof. Wood. *The London, Edinburgh, Dublin Philos. Mag. J. Sci.* **14**, 60–65, DOI: 10.1080/14786440709463661 (1907).
2. Wood, R. XLII. On a remarkable case of uneven distribution of light in a diffraction grating spectrum. *The London, Edinburgh, Dublin Philos. Mag. J. Sci.* **4**, 396–402, DOI: 10.1080/14786440209462857 (1902).
3. Khaidarov, E. *et al.* Asymmetric nanoantennas for ultrahigh angle broadband visible light bending. *Nano Lett.* **17**, 6267–6272, DOI: 10.1021/acs.nanolett.7b02952 (2017).
4. Ra'di, Y., Sounas, D. L. & Alù, A. Metagratings: Beyond the limits of graded metasurfaces for wave front control. *Phys. Rev. Lett.* **119**, 067404, DOI: 10.1103/PhysRevLett.119.067404 (2017).
5. Cariou, R. *et al.* III–V-on-silicon solar cells reaching 33% photoconversion efficiency in two-terminal configuration. *Nat. Energy* **3**, 326–333, DOI: 10.1038/s41560-018-0125-0 (2018).
6. Schygulla, P. *et al.* Two-terminal III–V//Si triple-junction solar cell with power conversion efficiency of 35.9% at AM1.5g. *Prog. Photovoltaics: Res. Appl.* 1–11, DOI: 10.1002/pip.3503 (2021).
